# InflaMix Predicts Severe CRS, ICU Admission, and Progression‐Free Survival After CAR‐T Therapy in LBCL

**DOI:** 10.1111/ejh.70307

**Published:** 2026-08-28

**Authors:** Eugenio Galli, Daniele Mannina, Alessandro Corrente, Chiara De Philippis, Ilaria Pansini, Marcello Viscovo, Stefan Hohaus, Patrizia Chiusolo, Federica Sorà, Stefania Bramanti, Simona Sica

**Affiliations:** ^1^ Department of Laboratory and Hematological Sciences Fondazione Policlinico Universitario A. Gemelli IRCCS Rome Italy; ^2^ Department of Hematology IRCCS Humanitas Research Hospital Rozzano Italy; ^3^ Hematology Section, Department of Radiologic and Hematologic Sciences Università Cattolica del Sacro Cuore Italy

## Abstract

**Objectives:**

Baseline systemic inflammation has emerged as a determinant of outcome after CD19‐directed CAR‐T therapy. We evaluated the prognostic performance of the recently developed INFLAmmation MIXture Model (InflaMix) in an independent bicentric cohort of patients with large B‐cell lymphoma (LBCL) receiving commercial CAR‐T cells, focusing on treatment‐related toxicities.

**Methods:**

We retrospectively analyzed 212 consecutive patients with relapsed/refractory LBCL treated with axi‐cel, tisa‐cel, or liso‐cel between 2020 and 2026. Patients were classified according to the InflaMix inflammatory phenotype. Progression‐free survival (PFS), CAR‐T‐related toxicities, ICU admission, and concordance with CAR‐HEMATOTOX and mEASIX were assessed.

**Results:**

An inflammatory InflaMix phenotype was identified in 21% of patients and was associated with inferior PFS (12‐month PFS: 30% vs. 56%; *p* < 0.001). While overall grade 2–4 CRS and ICANS rates were comparable, inflammatory patients showed higher rates of grade 3–4 CRS (11.1% vs. 1.8%; OR 3.18) and ICU admission (24.4% vs. 5.6%; OR 3.00). No association with ICAHT was observed. Concordance was poor with CAR‐HEMATOTOX and moderate with mEASIX.

**Conclusions:**

InflaMix was externally validated as a predictor of inferior PFS, severe CRS, and ICU admission after CAR‐T therapy, and candidates as a practical tool for pre‐infusion risk stratification and as a complement to existing scores.

## 1 Introduction

CD19‐directed chimeric antigen receptor T‐cell (CAR‐T) therapy has revolutionized the treatment of B‐cell non‐Hodgkin lymphomas (B‐NHL) and currently represents the preferred second‐line approach in relapsed/refractory large B‐cell lymphoma (LBCL), whenever feasible [1]. In this setting, complete and durable remission rates are approximately 40% [2], although practical tools for outcome prognostication are still needed to optimize treatment allocation and resource utilization.

A pre‐existing inflammatory state may impair immune effector cell function, alter the tumor microenvironment, and contribute to resistance to CAR‐T therapy [3, 4]. Pre‐treatment systemic inflammation has recently been recognized as a relevant determinant of outcomes also in other T‐cell–engaging therapies, including bispecific antibodies [5]. Accordingly, the identification of reliable biomarkers capturing baseline inflammatory status has gained increasing clinical interest. Among composite inflammation‐based scores, CAR‐HEMATOTOX and mEASIX have shown potential in predicting toxicity and refining outcome stratification [6, 7, 8, 9].

Beyond efficacy, safety remains a relevant concern in CAR‐T therapy, with non‐relapse mortality reported at approximately 6%–10% [10]. Major toxicities include cytokine release syndrome (CRS), immune effector cell‐associated neurotoxicity syndrome (ICANS), and immune effector cell‐associated hematotoxicity (ICAHT). Grade 2 CRS is typically managed in hematology wards, whereas grade 3–4 CRS often requires intensive care unit (ICU) transfer due to vasopressor and/or high‐flow oxygen support requirements. Patients developing severe CRS and ICAHT may experience increased mortality risk [11, 12].

In this context, Raj and colleagues developed InflaMix (INFLAmmation MIXture Model), a Gaussian mixture model based on 14 pre–CAR‐T infusion laboratory parameters reflecting inflammation, tumor burden, and organ function, accounting for inter‐variable covariance using routine laboratory data collected within 2 days before infusion [13]. The model stratifies patients into inflammatory and non‐inflammatory phenotypes in a binary fashion and remains applicable using a reduced panel of at least six variables. In patients with LBCL undergoing CAR‐T therapy, InflaMix was associated with progression‐free and overall survival, while showing no correlation with grade 2–4 CRS or ICANS.

## 2 Aim

In the present study, we applied InflaMix in a bicentric cohort of patients with LBCL treated with commercial CAR‐T products between 2020 and February 2026. The primary aim was to evaluate its ability to predict clinically relevant toxicities and to confirm its prognostic value for efficacy. Safety outcomes were CRS, ICANS, ICAHT, admission to the Intensive Care Unit (ICU), and requirement for treatment due to those events. Efficacy endpoints, namely PFS and OS, were also analyzed.

## 3 Patients and Methods

Inclusion criteria were being treated with commercial antiCD19 CAR‐T cells at Fondazione Policlinico Universitario A. Gemelli IRCCS (Rome) or Humanitas Research Hospital (Rozzano—Milan) between 2020 and 2025, being affected by aggressive large B‐cell lymphoma, and being aged 18+ years. CRS and ICANS were graded according to ASTCT Consensus [14], while ICAHT was graded as per current EHA/EBMT guidelines [15]. Patients were transferred to ICU whenever vasopressors or intensive treatments for respiratory distress were necessary. Events considered for PFS were any progression or evidence of refractory disease or death from any cause after CAR‐T infusion.

InflaMix was calculated according to the original model developed by Raj et al. using laboratory parameters obtained within 48 h before CAR‐T cell infusion through the algorithm reported online at https://github.com/vdblab/InflaMix [13]. InflaMix is an unsupervised Gaussian mixture model integrating 14 variables reflecting systemic inflammation, tumor burden, and organ function: hemoglobin, albumin, white blood cell count, platelet count, LDH, CRP, ferritin, D‐dimer, AST, ALP, total bilirubin, IL‐6, IL‐10, and TNF‐α. The model assigns patients to an inflammatory or non‐inflammatory cluster according to their probability of cluster membership. In the original study, the model could also be reliably applied using a simplified six‐parameter panel consisting of hemoglobin, albumin, LDH, CRP, AST, and ALP.

The modified Endothelial Activation and Stress Index (mEASIX) was calculated as LDH (U/L) × CRP (mg/dL)/platelet count (10^9^/L). mEASIX was developed as a modification of EASIX, replacing creatinine with CRP, and was proposed as a readily available marker of endothelial activation and stress, which are thought to contribute to the pathophysiology of severe CRS and ICANS after CAR‐T cell therapy [8]. In accordance with our previous findings, a baseline mEASIX value > 6.43 was considered high risk, as this threshold was identified by ROC analysis as predictive of grade ≥ 2 CRS (sensitivity 43%, specificity 100%, AUC 67%, *p* = 0.03) [9].

The CAR‐HEMATOTOX score was calculated according to the original model by Rejeski et al. using five pre‐lymphodepletion parameters reflecting hematopoietic reserve and systemic inflammation: platelet count, absolute neutrophil count, hemoglobin, CRP, and ferritin. One point was assigned for ANC ≤ 1200/μL, hemoglobin ≤ 9.0 g/dL, platelet count 76–175 × 10^9^/L, CRP ≥ 3.0 mg/dL, and ferritin 650–2000 ng/mL, while two points were assigned for platelet count ≤ 75 × 10^9/L and ferritin ≥ 2000 ng/mL^6^. Patients with a score of 0–1 were classified as low risk, whereas those with a score ≥ 2 were classified as high risk.

Comparisons among groups were conducted using descriptive statistics tools. Survivals were analyzed with Kaplan–Meier curves and Cox regression, while correlations were investigated with linear or logistic regressions, where indicated. Concordance between the binary risk classifications assigned by InflaMix, mEASIX, and CAR‐HEMATOTOX was assessed using cross‐tabulation and Cohen's kappa (*κ*) coefficient, with 95% confidence intervals. The proportion of concordant and discordant classifications was also reported, including the distribution of patients across concordant high‐ and low‐risk categories. κ values were interpreted according to conventional criteria, with higher values indicating greater agreement beyond chance. Data were censored in May 2026. Statistical analyses were performed using NCSS 2020 Statistical Software (NCSS LLC, Kaysville, UT, USA). All tests were two‐sided, and a *p* value < 0.05 was considered statistically significant. All participants provided informed consent for the anonymized use of their data. The study was conducted in accordance with the Declaration of Helsinki and was approved by the local ethics committee (CINECA ID4879; protocol V.4, Amendment 3, May/2026).

## 4 Results

Overall, 212 patients were enrolled. Patients received axi‐cel (54%), tisa‐cel (41%), or liso‐cel (5%), mostly as a second or third line of treatment; baseline characteristics are summarized in Table 1. The median age of the cohort was 61.5 years (range, 19–75), with 57.3% of patients aged ≥ 60 years, and 58.5% were male. Most patients had DLBCL (79.7%), whereas transformed follicular lymphoma (tFL) and primary mediastinal B‐cell lymphoma (PMBL) accounted for 16.5% and 3.8% of cases, respectively. Advanced‐stage disease (stage III–IV) was present in 82.8% of patients.

**TABLE 1:** Characteristics of the study population.

		Overall *N* = 212	Inflamix non inflammatory *N* = 167 (79)	Inflamix inflammatory *N* = 45 (21)	*p*
		*N* (95% C.I.) or *N* (%)			
Age	Median	61.5 (19–75)	62 (59–64)	59 (53–62)	0.181
	< 60 years	96 (45.3)	73 (76)	23 (24)	
	≥ 60 years	116 (57.3)	94 (81.1)	22 (18.9)	0.376
Gender	Females	88 (41.5)	67 (76.1)	21 (23.9)	
	Males	124 (58.5)	100 (80.6)	24 (19.4)	0.428
Histology	DLBCL	169 (79.7)	134 (79.3)	35 (20.7)	
	tFL	35 (16.5)	25 (71.4)	10 (28.6)	
	PMBL	8 (3.8)	8 (100)	0 (0)	0.190
Stage	I‐II	38 (17.9)	31 (81.6)	7 (18.4)	
	III‐IV	174 (82.8)	136 (78.2)	38 (21.8)	0.640
Previous lines	Median	2 (1–6)	2 (2–2)	2 (2–2)	
	1	64 (30.1)	54 (84.4)	10 (15.6)	
	2 or more	148 (69.9)	113 (76.3)	35 (23.7)	0.189
Costimulation	41BB	98 (46.2)	90 (78.9)	24 (21.1)	
	CD28	114 (53.8)	77 (78.6)	21 (21.4)	0.946
CAR‐HEMATOTOX	Low	70 (33)	53 (75.7)	17 (24.3)	
	High	142 (67)	114 (80.3)	28 (19.7)	0.444
mEASIX	< 6.43	168 (82.8)	145 (86.3)	23 (13.7)	
	≥ 6.43	35 (17.2)	14 (40)	21 (60)	< 0.001
Platelets ×10e3/uL	Median	138 (129–150)	136 (122–142)	147 (132–201)	0.059
Hemoglobin g/dL	Median	10.6 (10.5–10.9)	10.9 (10.6–11.11)	10.2 (9.7–10.8)	0.040
ANC cells/uL	Median	1400 (1547–1959)	1425 (1455–1801)	1320 (1412–2944)	0.123
LDH U/L	Median	203 (251–317)	193 (197–230)	410 (425–657)	< 0.001
CRP mg/L	Median	3.5 (14.8–33.2)	2.4 (8.2–18.6)	20.9 (25.5–100.8)	0.011
Ferritin ng/mL	Median	255 (351–558)	208 (298–479)	438 (282–1040)	0.164
CAR‐T peak cells/uL	Median	51.2 (31.0–106.5)	51.4 (46.1–94.2)	48.4 (15.8–68.3)	0.142
CRS max	Median	2 (range 0–4)	1 (range 0–3)	2 (range 0–4)	0.017
	Grade 3–4	8 (3.8%)	3 (1.8%)	5 (11.1%)	< 0.001
ICANS max	Median	0 (range 0–4)	0 (range 0–4)	0 (range 0–4)	0.281
ICU admission	Admitted	20 (9.7%)	9 (5.6%)	11 (24.4%)	< 0.001

*Note:* CAR‐HEMATOTOX and mEASIX see main text.

Abbreviations: ANC, absolute neutrophils count; CRP, C‐reactive protein; CRS, cytokine release syndrome; DLBCL, diffuse large B‐cell lymphoma; ICANS, immune effector cell‐associated neurotoxicity syndrome; ICU, intensive care unit; LDH, lactate dehydrogenase; PMBL, primary mediastinal B‐cell lymphoma; tFL, transformed follicular lymphoma.

InflaMix identified an inflammatory phenotype in 21% of patients, with no differences between the two centers (20% vs. 23%). Patients with an inflammatory profile had received a higher mean number of prior lines of therapy compared with non‐inflamed patients (2.1 vs. 1.8, *p* = 0.041), possibly reflecting more advanced disease biology or a greater impact of earlier treatment lines on systemic inflammation. The distribution of patients according to the number of previous treatment lines was otherwise comparable, with 69.9% of patients having received ≥ 2 prior lines of therapy. No significant differences were observed according to age, sex, histological subtype, disease stage, or CAR‐T product characteristics between inflammatory and non‐inflammatory patients.

The median follow‐up was 12 months (range 1–74). Overall progression‐free survival (PFS) was 67% (95% CI 61%–74%) at 3 months and 51% (95% CI 47%–61%) at 12 months. We observed no difference in efficacy outcomes among patients from the two centers (data not shown). CAR‐T efficacy was significantly inferior in patients with an inflammatory InflaMix profile, with PFS of 47% (95% CI 32%–62%) vs. 73% (95% CI 66%–79%) at 3 months and 30% (95% CI 16%–44%) vs. 56% (95% CI 48%–64%) at 12 months (*p* < 0.001) (Figure 1A). The cumulative incidence of relapse at 3 and 12 months was 51% (95% CI 38%–68%) and 68% (95% CI 55%–83%) in inflamed patients versus 26% (95% CI 20%–34%) and 42% (95% CI 35%–51%) in non‐inflamed patients, respectively (*p* < 0.001). The cumulative incidence of non‐relapse mortality at 3 and 12 months was 1% (95% CI 0.5%–4%) and 3% (95% CI 1.5%–7%), with no differences between groups (*p* = 0.931).

**FIGURE 1:**
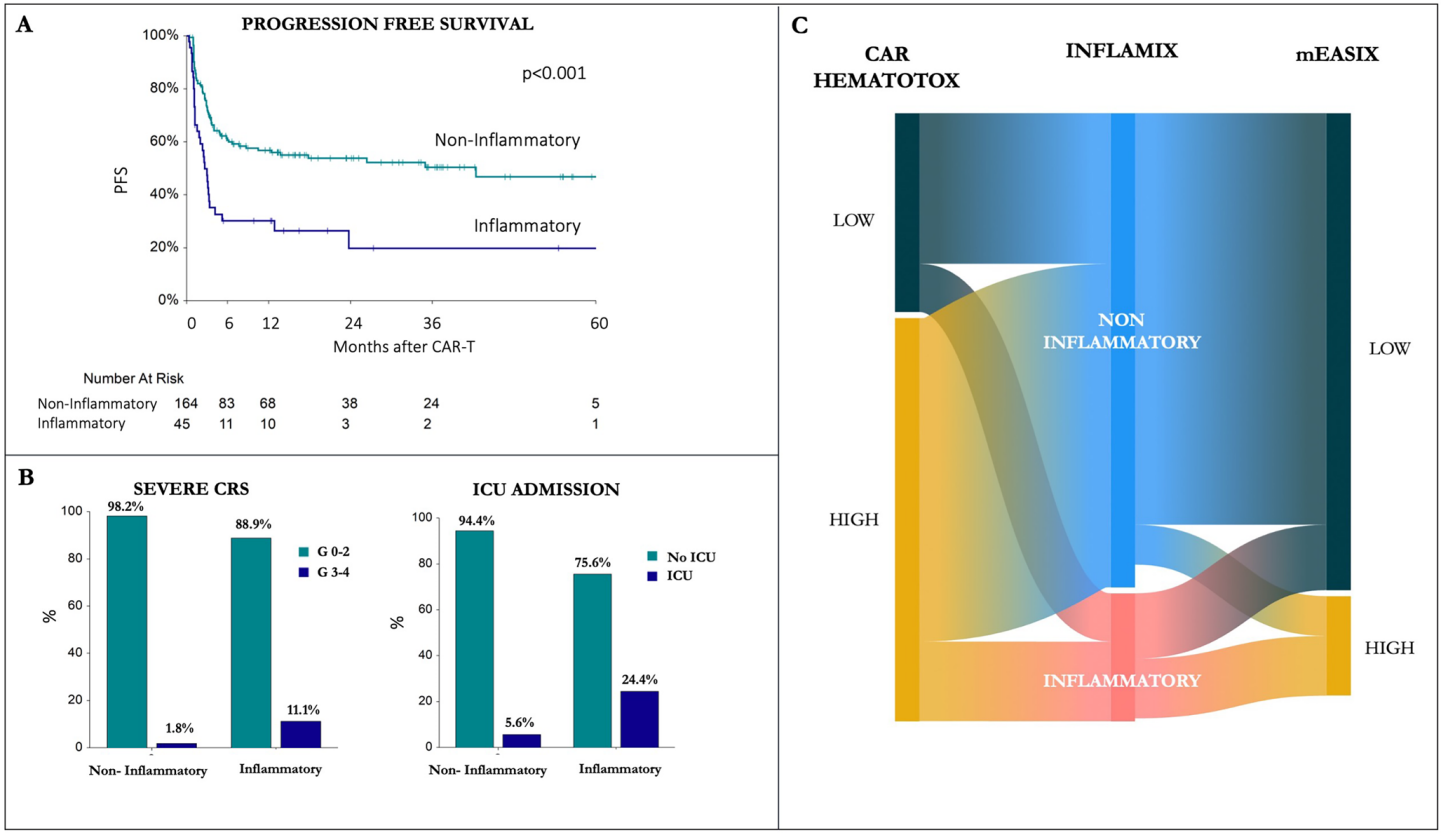
(A) Progression‐free survival according to InflaMix groups: Non‐inflammatory patients show better PFS compared to inflammatory patients (*p* < 0.001). (B) Inflammatory patients show a higher incidence of severe Grade 3–4 maximal CRS (11.%) and ICU admission (24.4%) compared to non‐inflammatory patients. (C) Concordance between CAR‐HEMATOTOX, mEASIX (cut‐off 6.43), and InflaMix. A low concordance between CAR‐HEMATOTOX and InflaMix was observed. CAR‐HEMATOTOX was low in 70 (33%) patients and high in 142 (77%) patients. Among patients with low CAR‐HEMATOTOX, 17 (25%) exhibited an inflammatory InflaMix profile, whereas 114 (80%) of patients with high CAR‐HEMATOTOX showed a non‐inflammatory InflaMix pattern. mEASIX classified 168 (83%) patients as low risk and 35 (17%) as high risk (9 missing values). Concordance between mEASIX and InflaMix was modest: Only 23/168 (13%) of low‐risk mEASIX patients were classified as inflammatory by InflaMix, while 14/35 (40%) of high‐risk mEASIX patients were classified as non‐inflammatory by InflaMix. These discrepancies suggest that, although the three scoring systems capture overlapping biological domains such as marrow reserve and systemic inflammation, they are not redundant but rather reflect distinct risk dimensions, thereby enabling complementary patient stratification.

Grade 2–4 CRS occurred in 51.4% of patients, with no significant difference between groups (48.5% vs. 62.2% in non‐inflammatory vs. inflammatory, respectively, *p* = 0.107). However, grade 3–4 CRS occurred more frequently in patients with an inflammatory profile (1.8% vs. 11.1%, OR 3.18, 95% CI 1.74–5.83, *p* < 0.001) (Figure 1B). Any‐grade ICANS occurred in 27% of patients and was grade 2–4 in 13%, with no significant differences between non‐inflamed and inflamed patients (any‐grade: 26.3% vs. 33.3%, *p* = 0.129; grade 2–4: 11.3% vs. 20%, *p* = 0.112, respectively).

Consistent with these findings, ICU admission was significantly higher in patients with an inflammatory InflaMix profile (5.6% vs. 24.4%, OR 3.00, 95% CI 1.82–4.95, *p* < 0.001) (Figure 1B). The overall ICU admission rate was 9.7%. The main indication for transfer to ICU was hypotension requiring at least 1 vasopressor. Tocilizumab was administered in 70% of patients, with no differences between InflaMix groups (68.2% vs. 77.7%, *p* = 0.228). Similarly, dexamethasone was administered in 38.8% of cases, without statistically significant differences between groups; however, steroid use was numerically higher among Inflammatory patients (46.6% vs. 36.6%, *p* = 0.221).

Early (Day 0–30) and late (Day 30–100) grade 3–4 ICAHT occurred in 30.9% and 8.7% of patients, respectively, with no significant differences between InflaMix groups (early: 30.9% vs. 31.1%, *p* = 0.979; late: 9.2% vs. 6.8%, *p* = 0.628). Patients with an inflammatory profile showed a non‐significant trend toward higher early infections (day 0–30) compared with non‐inflammatory patients (32.5% vs. 20.4%, *p* = 0.086).

To explore whether InflaMix captures overlapping or complementary biological features compared with established CAR‐T toxicity risk scores, we evaluated its concordance with CAR‐HEMATOTOX and mEASIX using predefined binary risk categories (Figure 1C, Tables 2A and 2B).

**TABLE 2A:** Cross‐classification of InflaMix with CAR‐HEMATOTOX and MEASIX risk categories.

Comparison		Comparator low	Comparator high	Total
*InflaMix versus CAR‐HEMATOTOX* (*n* = 212)				
InflaMix low	*n* (%)	53 (25.0%)	114 (53.8%)	167 (78.8%)
InflaMix high	*n* (%)	17 (8.0%)	28 (13.2%)	45 (21.2%)
Total	*n* (%)	70 (33.0%)	142 (67.0%)	212 (100%)
*InflaMix versus mEASIX* (*n* = 203)				
InflaMix low	*n* (%)	145 (71.4%)	14 (6.9%)	159 (78.3%)
InflaMix high	*n* (%)	23 (11.3%)	21 (10.3%)	44 (21.7%)
Total	*n* (%)	168 (82.8%)	35 (17.2%)	203 (100%)

**TABLE 2B:** Agreement between InflaMix and established CAR‐T toxicity scores.

Comparison	Concordant low‐risk	Concordant high‐risk	Overall agreement	Discordant cases	Cohen's kappa
InflaMix versus CAR‐HEMATOTOX (*n* = 212)	53 (25.0%)	28 (13.2%)	81 (38.2%)	131 (61.8%)	0.03
InflaMix versus mEASIX (*n* = 203)	145 (71.4%)	21 (10.3%)	166 (81.8%)	37 (18.2%)	0.42

Among 212 patients evaluable for both InflaMix and CAR‐HEMATOTOX, concordance was poor (kappa 0.03), with concordant classification observed in 81 patients (38.2%), including 53 patients (25.0%) classified as low risk by both models and 28 patients (13.2%) classified as high risk by both. Conversely, discordant risk assignment occurred in 131 patients (61.8%), mainly driven by patients categorized as high risk according to CAR‐HEMATOTOX but low risk according to InflaMix (114/212, 53.8%) (Figure 1C).

In contrast, a moderate degree of concordance was observed between InflaMix and mEASIX (*κ* = 0.42). Among 203 patients evaluable for both scores, concordant classification was observed in 166 patients (81.8%), including 145 patients (71.4%) classified as low risk and 21 patients (10.3%) classified as high risk according to both models. Discordant classifications occurred in 37 patients (18.2%), with the most frequent discordant pattern being InflaMix high/mEASIX low (23/203, 11.3%).

## 5 Discussion

In this paper, we confirm the ability of the InflaMix score, originally proposed by Raj and colleagues, to predict PFS after CAR‐T cells in LBCL, using an external cohort. in the original study. In their cohort, patients with an inflammatory phenotype had a significantly higher risk of progression or death (HR 2.98, 95% CI 1.60–4.91), and this association remained significant after adjustment for established prognostic factors, including tumor burden. In our cohort, patients with an inflammatory phenotype also had significantly worse PFS, with 3‐ and 12‐month PFS rates of 47% and 30%, respectively, compared with 73% and 56% in patients with a non‐inflammatory phenotype. Although direct comparison of survival estimates between the two studies should be made with caution because of differences in patient populations and treatment characteristics, the consistency of these findings supports the prognostic value of InflaMix in patients with LBCL undergoing CAR‐T therapy. In addition, our results suggest that the application if InflaMix could be extended for prediction of severe toxicities. Consistent with the original report by Raj et al., we observed no association with grade 2–4 CRS; however, we decided to focus on clinically severe toxicities, finding that patients with an inflammatory profile showed a markedly increased risk of grade 3–4 CRS and a substantially higher ICU admission rate (24.4% vs. 5.6%), an association that was not observed in the original study.

The use of tocilizumab and steroids was similar among groups. In some cases, treatment of CRS or ICANS was administered in an early‐intervention strategy, in line with emerging evidence from ZUMA‐1 cohorts 4 and 6 [16]. Such “early intervention” approaches may partly explain the absence of significant differences between groups for these endpoints concerning grade 2–4 CRS or ICANS. Conversely, very high grade CRS (Grade 3–4) was augmented in patients with an inflammatory profile, suggesting a possible impact of highly inflammatory milieu or massive tumor burden on CRS.

Overall, several clinical and laboratory parameters have been investigated and combined as predictors of outcome after CAR‐T therapy, including disease burden, LDH, inflammatory markers such as CRP and ferritin, response to bridging therapy, and characteristics of the CAR‐T product [7, 17, 18, 19]. However, none of these parameters have emerged as a single, sufficiently robust predictor of treatment efficacy across different studies and patient populations. Importantly, the original study by Raj et al. showed that InflaMix retained prognostic value even after accounting for tumor burden, suggesting that the score captures information that is not simply explained by the amount of disease. Currently available predictive scores capture different aspects of the patient's condition before CAR‐T infusion. CAR‐HEMATOTOX mainly reflects hematopoietic reserve and systemic inflammation and was initially developed to predict hematologic toxicity, although its association with PFS and OS has also been subsequently reported [7]. mEASIX, on the other hand, was developed to capture endothelial activation and stress and has mainly been investigated in relation to CRS and ICANS [8, 9]. In this context, InflaMix may provide additional information rather than simply representing an alternative to these scores. Concerning concordance among predictive risk scores, our findings indicate that InflaMix shares partial overlap with mEASIX, consistent with their common ability to capture systemic inflammatory and endothelial activation pathways, while showing limited concordance with CAR‐HEMATOTOX, suggesting that these models capture different biological aspects of CAR‐T–related toxicity. The poor concordance between InflaMix and CAR‐HEMATOTOX (*κ* = 0.03) supports this hypothesis, whereas the moderate concordance observed with mEASIX (*κ* = 0.42) may reflect a greater overlap in the inflammatory and endothelial pathways represented by the two models. Overall, these findings suggest that current scores capturing inflammatory activation, endothelial dysfunction, and hematopoietic vulnerability should be considered complementary rather than interchangeable tools for assessing CAR‐T toxicity risk.

InflaMix is derived from pre‐infusion laboratory parameters and therefore is not intended as a standalone tool for CAR‐T eligibility decisions. Nevertheless, several practical implications can be derived:

1. Optimal scheduling and toxicity management: High‐risk patients may benefit from intensified monitoring and proactive ICU alert, given that approximately one in four require ICU admission, whereas a less restrictive ICU allocation strategy may be reasonable in low‐risk patients. Beyond predicting toxicity, the early identification of patients at increased risk of ICU admission after CAR‐T therapy has important clinical implications. A reliable risk stratification approach may facilitate appropriate resource allocation, support treatment setting decisions (including the selection of patients suitable for outpatient administration or early discharge strategies), and enable closer monitoring of individuals at highest risk. This is particularly relevant considering that ICU admission after CAR‐T has been associated with increased non‐relapse mortality, as reported by Azoulay et al., highlighting the potential impact of early risk assessment on patient management and outcomes [11].
2. Patients with high InflaMix scores may benefit from prophylactic strategies, including corticosteroid prophylaxis and potentially anakinra, to mitigate the risk of severe CRS.
3. High‐InflaMix patients should undergo early reassessment at 1 month, given their increased risk of non‐response.

Finally, InflaMix may serve as a useful benchmark for evaluating bridging therapy efficacy, as it reflects tumor‐associated inflammatory burden. Reducing this inflammatory component may represent a key objective of optimal bridging strategies aimed at improving CAR‐T efficacy.

## Author Contributions

E.G., D.M., S.B., and S.S. conceived the paper. E.G. and A.C. drafted the paper. I.P., D.M., C.D.P., A.C., F.S. collected data. D.M., C.D.P., E.G., S.B., M.V., S.H., P.C., F.S., and S.S. managed patients. All authors revised and approved the final manuscript.

## Funding

The authors have nothing to report.

## Conflicts of Interest

The authors declare no conflicts of interest.

## Data Availability

The data that support the findings of this study are available from the corresponding author upon reasonable request.
